# Psychotic symptoms in tyrosine hydroxylase deficiency-associated dopa-responsive dystonia treated with antipsychotics and rTMS: a case report

**DOI:** 10.3389/fpsyt.2026.1920048

**Published:** 2026-10-08

**Authors:** Yue Shen, Hai-Zhi Chen, Liang Lv, Lan-lan Fang

**Affiliations:** Department of Psychiatry, Huzhou Third Municipal Hospital, Huzhou, Zhejiang, China

**Keywords:** case report, dopamine-responsive dystonia, repetitive transcranial magnetic stimulation (rTMS), schizophrenia, tyrosine hydroxylase deficiency

## Abstract

**Background:**

Tyrosine hydroxylase deficiency (THD) is a rare autosomal recessive disorder caused by mutations in the *TH* gene. THD encompasses a broad phenotypic spectrum that includes dopamine-responsive dystonia (DRD) as its milder presentation, alongside severe infantile parkinsonism and progressive infantile encephalopathy. Although non-motor manifestations—such as depression, anxiety, and sleep disturbances—have garnered increasing attention, psychotic symptoms including hallucinations and delusions remain rarely reported in THD.

**Case summary:**

This report describes a genetically confirmed woman with THD who presented with infantile-onset dystonia. Auditory hallucinations and erotomanic delusions emerged approximately five years after levodopa initiation and persisted despite one-month levodopa withdrawal. Although levodopa markedly improved her dystonia, antipsychotic treatment posed a therapeutic dilemma: risperidone exacerbated motor symptoms, and aripiprazole was limited by poor adherence. A multimodal regimen comprising aripiprazole, quetiapine, and low-frequency repetitive transcranial magnetic stimulation (rTMS) targeting the left temporoparietal cortex was associated with progressive improvement of psychotic symptoms without clinically apparent worsening of dystonia. The independent contribution of rTMS could not be determined given the concurrent pharmacological titration.

**Conclusion:**

Selecting appropriate antipsychotic medication in THD patients with prominent psychotic symptoms poses a considerable challenge. The combination of aripiprazole, quetiapine, and rTMS may represent a potential therapeutic option. To our knowledge, this is the first reported case of genetically confirmed DRD due to THD presenting with prominent psychotic symptoms, and among the few documenting rTMS use in this population. These observations are based on clinical assessment without standardized quantitative measures; the independent efficacy of rTMS cannot be established from this case.

## Introduction

Tyrosine hydroxylase deficiency (THD) is a rare autosomal recessive disorder caused by mutations in the *TH* gene, with an estimated prevalence of 1–9 per million (1). Mutations in this gene result in reduced synthesis of dopamine, norepinephrine, and epinephrine, producing a broad phenotypic spectrum that ranges from milder dopamine-responsive dystonia (DRD) to severe infantile parkinsonism with bradykinesia and progressive infantile encephalopathy (2, 3). TH-deficient dopamine-responsive dystonia (DRD) is characterized by lower-limb-onset dystonia and gait difficulty, with onset typically occurring between 12 months and 12 years of age; diurnal fluctuation, with symptoms typically worsening toward the evening and improving after sleep, is a hallmark feature of DRD. Levodopa remains the first-line treatment (4, 5). Severe and very severe forms—referred to as TH-deficient infantile parkinsonism with bradykinesia and TH-deficient progressive infantile encephalopathy, respectively—typically manifest earlier, often in the neonatal period or shortly after birth. Clinical features include truncal hypotonia, bradykinesia, rigidity, and other parkinsonian signs, frequently accompanied by ptosis, intermittent somnolence, hyperreflexia, and intellectual disability. These forms respond slowly to levodopa and are associated with poor drug tolerability (6).

In recent years, non-motor manifestations of DRD/THD have garnered increasing recognition; common psychiatric comorbidities include sleep disturbances, anxiety, depression, panic attacks, and obsessive-compulsive behaviors (7–9). Nevertheless, cases presenting with psychotic symptoms such as auditory hallucinations and delusions are exceedingly rare. Only a handful of case reports have described DRD patients with comorbid schizophrenia spectrum disorders (10–12). Because dopaminergic medications and antipsychotics exert opposing effects, managing such cases presents a formidable challenge. This report describes the multimodal management of a patient with THD and comorbid psychotic symptoms, aiming to inform clinical decision-making for similarly complex cases.

## Case presentation

### Chief complaints

A 28-year-old woman with infantile-onset dystonia was admitted for management of persistent auditory hallucinations and erotomanic delusions of 8 years’ duration.

### History of present illness

At admission, the patient exhibited prominent psychotic symptoms, including auditory hallucinations, persecutory delusions, and erotomanic delusions. She displayed marked emotional lability, with prominent irritability, impulsive spending, and sleep disturbances. The patient reported that her mood fluctuations were closely tied to the content of the auditory hallucinations: distress intensified when the voices were derogatory or threatening, whereas neutral or seemingly supportive content provided transient relief. Impulsive spending was primarily driven by the erotomanic delusion (e.g., purchasing gifts for its object). Notably, psychotic symptoms remained evident even during periods of relative emotional stability. The patient repeatedly contacted and harassed her supervisor due to the erotomanic delusion, resulting in severe occupational impairment. She also exhibited progressive social withdrawal, emotional blunting toward family members, and a pervasive sense of insecurity in interpersonal relationships.

The patient developed an abnormal gait at age 1. By age 2, she experienced intermittent episodes characterized by an inability to lift her head, sit upright, or walk, each lasting several minutes before spontaneous resolution. She underwent multiple evaluations in the neurology department; cranial MRI and EEG revealed no significant abnormalities, and no pharmacotherapy was initiated at that time.

At age 6, she developed dystonia that initially involved the right lower limb, accompanied by dysphagia, gait instability, dystonic posturing, and periorbital and perioral involuntary movements. After initiating levodopa (125 mg three times daily) at age 15, her dystonia improved markedly. Additionally, the patient began experiencing prominent auditory hallucinations at age 20, consisting primarily of negative, critical voices, accompanied by low mood, irritability, hypersensitivity, suspiciousness, outbursts of anger, and erotomanic delusions. Of note, the dosage of levodopa had not been adjusted prior to the onset of these psychiatric symptoms.

At age 23, she first presented to a psychiatric clinic. After treatment with low-dose risperidone, her psychiatric symptoms failed to improve, and her dystonia worsened. A switch to aripiprazole led to significant improvement in her auditory hallucinations and delusions, with concurrent mood stabilization. At age 24, hallucinations and delusions recurred intermittently due to poor medication adherence. She subsequently attempted to discontinue levodopa in an effort to control her psychiatric symptoms. After approximately one month of withdrawal, her psychiatric symptoms showed no remission, whereas dystonia markedly worsened, prompting resumption of levodopa. At age 27—six months prior to the current admission—her auditory hallucinations and delusions markedly worsened after complete discontinuation of aripiprazole. In the same year, whole-exome sequencing confirmed a diagnosis of THD manifesting as DRD. Molecular genetic testing identified compound heterozygous mutations in the *TH* gene: c.694C>T and c.739G>A (chromosome 11q15.5).

Despite unsupervised upward titration of aripiprazole to 30 mg/day in the week prior to admission, her psychiatric symptoms remained refractory.

### Past medical history

The patient had attempted suicide once by drug overdose at age 27, precipitated by distress from auditory hallucinations. She had also undergone bilateral lower-extremity tendon-lengthening surgery during childhood for dystonia. She denied any other significant medical conditions or additional surgical history.

### Personal and family history

The patient denied tobacco or alcohol use, illicit drug abuse, or drug allergies. No family history of similar conditions was reported.

### Physical examination

Vital signs were stable. Under levodopa therapy, the motor examination was essentially normal: speech was clear and fluent; gait was normal; eye movements were full without oculogyric crises; muscle strength was normal in all four limbs; muscle tone was normal; and no drug-induced dyskinesia was observed. These findings are highly consistent with the classic treatment response of dopa-responsive dystonia (DRD) due to tyrosine hydroxylase deficiency.

### Mental status examination

The patient was alert and cooperative, responded appropriately to questions, and demonstrated intact memory, orientation, and calculation abilities. She experienced auditory hallucinations, erotomanic delusions, and persecutory delusions. Emotional lability was secondary to these psychotic symptoms, with associated impulsive behavior.

### Ancillary tests

Ancillary tests were performed to exclude secondary causes of psychotic symptoms and movement disorders, including structural brain lesions and metabolic encephalopathies. Head CT and chest CT revealed no abnormalities. No abnormalities were found in the complete blood count, liver and kidney function tests, blood glucose, lipid profile, or thyroid function tests.

## Treatment

At admission, the patient reported frequent derogatory auditory hallucinations (approximately 20–30 times daily), along with erotomanic delusions (firmly believing that her supervisor harbored romantic feelings for her) and persecutory delusions (perceiving that people around her intended to harm her). Her mood was notably irritable.

After admission, levodopa was continued at 125 mg three times daily to control dystonia.

For psychiatric pharmacotherapy, aripiprazole was maintained at 30 mg/day based on the patient’s prior favorable response and the recent titration to this dose during the week preceding admission, while quetiapine was initiated at 25 mg/day and titrated upward by 50 mg every 3 days, reaching 300 mg/day by hospital day 14. Adjunctive low-frequency repetitive transcranial magnetic stimulation (rTMS) was initiated on hospital day 7 for refractory auditory hallucinations, delivered using an NTK-TMS-II device (Jiangxi Brain Modulation Technology Development Co., Ltd.) with a figure-of-eight coil positioned over the left temporoparietal cortex (T3-P3) according to the International 10–20 EEG electrode placement system. Stimulation parameters were as follows: 1 Hz at 80%–90% of the resting motor threshold; 25 minutes per session, 1,500 pulses per session, for a total of 10 sessions over 2 consecutive weeks (5 sessions per week), yielding a cumulative total of 15,000 pulses.

On day 21, the patient reported that auditory hallucinations had decreased to 5–6 times daily, with markedly reduced intensity; however, her erotomanic and persecutory delusions persisted, and she continued to exhibit emotional lability and poor sleep quality. Clonazepam 1 mg was therefore added at bedtime to improve nocturnal sleep. Between days 30 and 50, the patient experienced fluctuating auditory hallucinations, with occasional irritability, angry outbursts, and suspiciousness toward others in the ward, accompanied by a belief that someone might intend to harm her. During this period, quetiapine was gradually titrated upward, reaching 600 mg/day (in two divided doses) by hospital day 50. Throughout the treatment period, the frequency of auditory hallucinations was assessed based on patient self-reports and observations by clinicians and nursing staff. The complete medication titration process is summarized in **Table 1**.

**Table 1:** Medication titration and clinical course during hospitalization.

Time point	Levodopa	Aripiprazole	Quetiapine	Clonazepam	Clinical notes
Admission (Day 1)	125 mg tid	30 mg/day	25 mg/day	—	Auditory hallucinations (20–30 times/day), poor sleep, erotomanic and persecutory delusions; no significant dystonia.
Day 7	125 mg tid	30 mg/day	150 mg/day	—	Sleep improved, but hallucinations and delusions persisted; adjunctive rTMS initiated.
Day 14	125 mg tid	30 mg/day	300 mg/day	—	Hallucinations reduced to 15 times/day with decreased intensity.
Day 21	125 mg tid	30 mg/day	300 mg/day	1 mg qn	The 10-session rTMS course was well tolerated, with no headache, dizziness, or other adverse effects reported. Sleep worsened (4 hours/night), and hallucinations decreased to 5–6 times/day with markedly reduced intensity.
Day 28	125 mg tid	30 mg/day	400 mg/day	1 mg qn	Hallucinations 5–8 times/day; erotomanic delusions prominent, repeatedly contacted the individual she believed had romantic feelings for her. Sleep duration increased to 6 hours/night.
Day 35	125 mg tid	30 mg/day	450 mg/day	1 mg qn	Hallucinations 3–5 times/day; temper outbursts in the ward; suspicious that others intended to harm her.
Day 42	125 mg tid	30 mg/day	500 mg/day	1 mg qn	Hallucinations fluctuated (3–10 times/day); mood occasionally unstable; hostile toward her teacher, sending insulting messages.
Day 50	125 mg tid	30 mg/day	600 mg/day	1 mg qn	Hallucinations 1–3 times/day; suspiciousness reduced; erotomanic delusions persisted.
Day 51–60 (Discharge)	125 mg tid	30 mg/day	600 mg/day	1 mg qn	Hallucinations virtually resolved; mood stabilized; no longer suspicious of others; erotomanic delusions subsided; no longer pestering others; no significant sedation, dystonia, or other adverse effects observed.

qn., every night; rTMS, repetitive transcranial magnetic stimulation; tid, three times daily. A dash (—) indicates that the medication was not used at that time point.

All doses are expressed as total daily doses unless otherwise specified. Levodopa 125 mg tid was maintained throughout the entire hospitalization for dystonia control and continued at discharge.

## Outcome and follow-up

At admission, the patient’s psychiatric baseline was characterized by frequent derogatory auditory hallucinations (20–30 times daily), persistent erotomanic and persecutory delusions, and marked irritability. The motor baseline on levodopa 375 mg/day plus aripiprazole 30 mg/day showed no clinically apparent dystonia or extrapyramidal symptoms on examination. From hospital day 51 to day 60, based on patient self-report and clinical observation, her auditory hallucinations almost completely resolved, with overall mood improvement. She no longer suspected that others intended to harm her, and her erotomanic delusions markedly subsided. Throughout the two-month hospitalization, no sedation, weight gain, or extrapyramidal symptoms were observed clinically. At the 1-month follow-up after discharge, her psychiatric symptoms continued to remit and motor function remained stable.

## Discussion

Clinically, THD encompasses a phenotypic spectrum ranging from DRD to TH-deficient infantile parkinsonism with bradykinesia and TH-deficient progressive infantile encephalopathy. Recent consensus guidelines emphasize phenotype-driven classification based on age at onset, disease progression, and treatment response, rather than rigid severity-based grading (1). The combination of infantile-onset dystonia, marked levodopa responsiveness, and preserved cognitive function supports the diagnosis of THD manifesting as DRD.

The diagnosis of mental illness represents a central challenge in this case. Over an 8-year illness course, the patient presented with prominent psychotic symptoms—primarily hallucinations and delusions—accompanied by marked impairment in social and occupational functioning. Although affective symptoms such as emotional outbursts, depressed mood, or irritability were observed during the course of illness, these were secondary to the psychotic symptoms and of brief duration; psychotic symptoms persisted even during periods of relative emotional stability. There is currently insufficient evidence to support the presence of an independent major depressive episode or manic episode meeting the diagnostic criteria of the *Diagnostic and Statistical Manual of Mental Disorders, Fifth Edition* (DSM-5). Consequently, schizoaffective disorder, as well as major depressive disorder or bipolar disorder with psychotic features, are considered less likely.

Meanwhile, it should not be overlooked that long-term levodopa use itself may also induce psychiatric symptoms. Clinically, levodopa serves as a first-line treatment for Parkinson’s disease, and approximately 10%–15% of patients develop psychiatric disorders during the course of treatment (13), with hallucinations and delusions being the most common manifestations. The dopamine hyperfunction hypothesis provides an important pathophysiological basis for explaining levodopa-induced psychotic symptoms: as a dopamine precursor, levodopa enhances dopaminergic neurotransmission in the brain, particularly within the mesolimbic system, thereby triggering psychotic symptoms. Furthermore, some studies suggest that serotonergic mechanisms may also play a significant role in this process, which may account for the sleep disturbances and mood problems observed in Parkinson’s disease patients receiving levodopa therapy (14). In our patient, the emergence of psychotic symptoms was not preceded by a significant increase in levodopa dosage, and these symptoms persisted for approximately one month after levodopa discontinuation. These observations suggest that the aforementioned manifestations are more likely attributable to a comorbid psychiatric disorder or underlying dopaminergic dysfunction per se, rather than an acute adverse reaction to dopaminergic therapy. However, these observations do not entirely exclude a modulatory role of chronic dopaminergic treatment in the onset or severity of psychotic symptoms. Some researchers have proposed that long-term levodopa use may lead to intermittent surges in dopamine levels, resulting in sensitization of the mesolimbic pathway and subsequent induction of psychotic symptoms (15).

Although motor symptoms remain the cardinal feature of THD, non-motor manifestations have garnered increasing recognition in recent years. The consensus guideline for THD emphasizes that a multidisciplinary treatment approach should be utilized to monitor neurocognitive development and other comorbidities that may occur in THD. However, the spectrum of psychiatric symptoms in THD remains incompletely characterized.

Anxiety and depression have been reported in THD patients. García Alarcón et al. described a young woman with genetically confirmed THD who developed severe panic disorder and major depression around the time of onset of her movement disorder (9). Notably, while her motor symptoms responded to levodopa, her anxiety did not improve with dopaminergic therapy alone and ultimately required the addition of a serotonergic antidepressant (escitalopram), suggesting that psychiatric symptoms in THD may involve neurotransmitter systems beyond dopamine. This observation is particularly relevant to our patient, whose psychotic symptoms likewise failed to remit with levodopa adjustment alone and required targeted antipsychotic and neuromodulatory interventions.

Psychotic symptoms, including hallucinations and delusions, are considerably rarer in THD than affective symptoms. Parikh and Panse reported a case of DRD complicated by schizophrenia in a 32-year-old male with a 20-year history of dystonia and 16-year history of psychotic symptoms (11). The patient was successfully treated with levodopa 250 mg twice daily for dystonia and clozapine 200 mg for psychotic symptoms. However, genetic testing was not performed in that case, leaving the causative gene unidentified. In contrast, our patient’s diagnosis was genetically confirmed by whole-exome sequencing, which identified compound heterozygous mutations in the *TH* gene (c.694C>T and c.739G>A), providing definitive etiological clarification.

Another relevant report by Wang et al. described a 20-year-old male patient with probable dopa-responsive dystonia complicated by schizoaffective disorder, who presented with both dystonia and psychotic symptoms including auditory hallucinations and persecutory delusions after a period of medication noncompliance (10). In that case, low-dose risperidone was added to levodopa therapy, combined with benztropine to alleviate dystonia. The psychiatric symptoms improved, but genetic testing was not performed, leaving the causative genes unidentified. In contrast, our case provides genetically confirmed THD with clear documentation of both the movement disorder and the psychiatric phenotype, as well as a detailed account of the treatment course.

THD leads to reduced dopamine synthesis in the nigrostriatal and mesolimbic pathways (1, 16). Dopamine deficiency in the striatum causes dystonia, whereas the relationship between mesolimbic dopamine and psychosis is more complex. The classical model posits that excessive subcortical dopamine leads to psychotic symptoms (17); however, THD is characterized by generalized dopamine deficiency.

Although dopaminergic dysfunction constitutes the core pathophysiological basis of motor symptoms in THD, its psychiatric comorbidities may originate from broader monoaminergic neurotransmitter system disturbances. As the rate-limiting enzyme in catecholamine (dopamine and norepinephrine) synthesis, tyrosine hydroxylase deficiency leads to decreased levels of homovanillic acid (HVA) and 3-methoxy-4-hydroxyphenylglycol (MHPG) in cerebrospinal fluid, reflecting impaired central dopaminergic and noradrenergic neurotransmission. Of particular importance, the characteristic CSF biochemical profile in THD is marked by low HVA, low MHPG, normal 5-hydroxyindoleacetic acid (5-HIAA) levels, and a significantly reduced HVA/5-HIAA ratio (1). Specifically, the markedly reduced HVA/5-HIAA ratio may reflect a dysregulated state between dopaminergic and serotonergic systems. In addition, generalized dopamine deficiency may lower the threshold for psychotic symptoms through compensatory postsynaptic D_2_ receptor upregulation or serotonergic dysfunction (2, 18). This distinctive pattern of “dopamine-norepinephrine deficiency with relative serotonin preservation” suggests that THD-associated psychotic symptoms do not arise solely from dopamine deficiency, but may instead stem from functional imbalance among multiple neurotransmitter systems (19). This pattern parallels other neurotransmitter disorders, such as GTP cyclohydrolase 1 deficiency and aromatic L-amino acid decarboxylase deficiency, in which psychiatric manifestations are increasingly recognized as integral components of the phenotype (18). Previous reports demonstrating the efficacy of serotonergic antidepressants for anxiety and depressive symptoms in THD patients further support the involvement of serotonergic dysfunction (9). In our patient, the persistence of psychotic symptoms after levodopa discontinuation, together with the ultimate requirement for combined antipsychotic and neuromodulation therapy, suggests that non-dopaminergic mechanisms played a critical role in her psychiatric comorbidities.

The *TH* gene itself has been investigated as a candidate gene in schizophrenia. While some studies have explored associations between *TH* gene polymorphisms and schizophrenia, linkage analyses have not consistently supported a direct genetic link. The co-occurrence of THD and schizophrenia-like psychopathology in our patient may reflect independent genetic vulnerability, pleiotropic effects of *TH* gene mutations, or complex interactions between dopaminergic dysfunction and other neurodevelopmental factors. Consequently, although the patient exhibited clinically characteristic features of schizophrenia, the precise contribution of THD to her psychiatric manifestations could not be established in this case and awaits further elucidation.

For patients with THD and concurrent schizophrenia-like features, pharmacological management presents a significant therapeutic dilemma. Levodopa is essential for controlling the motor symptoms of DRD, but its excessive dopaminergic stimulation of the mesolimbic pathway may induce or exacerbate psychotic symptoms—a phenomenon well recognized in Parkinson’s disease (20). Conversely, antipsychotic medications (particularly first-generation and some second-generation drugs) block dopamine D_2_ receptors, which may worsen dystonia, rigidity, and bradykinesia in patients who already have dopaminergic deficits (1, 2).

In choosing medication for this patient, we first excluded drugs with high D_2_ receptor affinity (such as risperidone). The patient’s history of worsened dystonia following low-dose risperidone further supports this rationale. We decided to continue aripiprazole for the following reasons. First, aripiprazole is a partial dopamine D_2_ receptor agonist that stabilizes rather than completely blocks dopaminergic transmission, which may ameliorate psychiatric symptoms while mitigating severe dopamine depletion (21). In our patient, this agent had previously demonstrated favorable efficacy and tolerability. However, it should be noted that motor worsening has been reported with aripiprazole in other disorders characterized by dopaminergic dysfunction (22, 23); therefore, its partial D_2_ agonist profile should not be construed as conferring universal motor safety. Second, quetiapine has low D_2_ receptor affinity (24), is widely used in Parkinson’s disease psychosis, and is associated with a relatively low risk of motor deterioration (25). Currently, evidence to guide antipsychotic selection in THD remains extremely limited. The therapeutic regimen in this case was therefore formulated primarily based on extrapolation from evidence in other disorders, together with the patient’s individual clinical response.

Furthermore, the patient received low-frequency repetitive transcranial magnetic stimulation (rTMS) as an adjunctive treatment. The stimulation protocol employed a standard figure-of-eight coil, with the target localized to the left temporoparietal cortex (T3-P3 based on the International 10–20 EEG electrode placement system)—a region established as the classic target for auditory hallucinations (26). Resting motor threshold (RMT) was determined through visual observation with the target muscle in a state of complete relaxation, defined as the minimum stimulus intensity required to elicit visible contraction of the target muscle in at least 5 out of 10 consecutive stimuli (27). According to the Operational Guidelines for the Clinical Application of Repetitive Transcranial Magnetic Stimulation in Mental Disorders published by the Chinese Medical Education Association, the recommended stimulation intensity for auditory hallucinations ranges from 80% to 100% of RMT. In this case, based on individualized tolerability assessment, the stimulation intensity was set at 80%–90% of RMT. The treatment parameters were as follows: stimulation frequency of 1 Hz, 1,500 pulses per session, for a total of 10 sessions, yielding a cumulative total of 15,000 pulses. rTMS is a non-invasive neuromodulation intervention for treatment-resistant auditory hallucinations (26); its mechanism of action involves modulation of excitability and functional connectivity in language-processing regions, rather than direct dopaminergic effects. To the best of our knowledge, this represents one of the few reports documenting rTMS use in a patient with THD. Notably, preclinical studies have demonstrated that low-frequency rTMS can enhance tyrosine hydroxylase (TH) expression in dopaminergic neurons and exert neuroprotective effects in animal models of Parkinson’s disease (28). The findings in this case support the feasibility and short-term tolerability of adjunctive rTMS—no headache, dizziness, or other adverse events were observed over the 10-session course. However, given that quetiapine was undergoing rapid dose titration concurrently with rTMS, the independent contribution of rTMS to psychiatric symptom improvement could not be determined in this patient.

The decision to initiate rTMS concurrently with quetiapine titration, rather than after a formal observation period, was driven by clinical urgency and safety considerations. The patient’s psychotic symptoms were severe and persistent—auditory hallucinations occurring 20–30 times daily with marked occupational and social impairment—and had worsened markedly after aripiprazole discontinuation, remaining refractory despite unsupervised upward titration of aripiprazole to 30 mg/day in the week before admission. Her history of a suicide attempt precipitated by hallucinatory distress further underscored the need for rapid symptom control. Because quetiapine had never been trialed and required gradual dose escalation over weeks, whereas rTMS could be initiated immediately without systemic dopaminergic effects and with minimal risk of motor deterioration, the combined approach was adopted to accelerate symptom relief.

Of note, the patient tolerated quetiapine at 600 mg/day during short-term clinical follow-up, without clinically detectable sedation, weight gain, or extrapyramidal symptoms. This finding is of considerable clinical significance, as higher quetiapine doses are associated with increased D_2_ receptor occupancy, which may theoretically exacerbate motor symptoms in patients with pre-existing dopaminergic deficits. However, given that no standardized motor assessments were conducted in this case and the post-treatment follow-up was limited to one month, this observation should not be extrapolated to the safety profile or therapeutic window of quetiapine in the broader THD population.

This case offers several clinically relevant lessons. First, clinicians should maintain a high index of suspicion for psychotic disorders in patients with THD. While anxiety and depression are increasingly recognized in this population, psychotic symptoms may be underrecognized and undertreated. Second, when antipsychotic therapy is indicated in THD patients, agents with low D_2_ receptor affinity (e.g., quetiapine) or partial D_2_ receptor agonists (e.g., aripiprazole) may be considered; however, given that evidence guiding antipsychotic selection in THD remains extremely limited, pharmacological strategies should be individualized under close monitoring. Furthermore, rTMS may serve as an adjunctive strategy for treatment-resistant auditory hallucinations in THD, offering a non-pharmacological intervention that circumvents systemic dopaminergic effects; nevertheless, its independent efficacy cannot be determined from this single case.

Several limitations of this case report should be acknowledged. First, this is a single case report, and the findings may not be generalizable to other THD patients. Second, standardized psychiatric rating scales (such as PANSS or BPRS) and motor rating scales (such as UPDRS or BFMDRS) were not systematically administered, limiting the quantitative assessment of treatment response. The frequency of auditory hallucinations was evaluated through patient self-report and clinical observation rather than structured instruments; motor outcome assessment was subject to the same methodological limitation. Third, the follow-up duration was limited (only one month post-discharge), precluding assessment of the durability of psychiatric symptom improvement as well as the long-term motor tolerability and metabolic safety of high-dose quetiapine. Fourth, because rTMS was administered concurrently with rapid quetiapine titration, its independent contribution to clinical improvement could not be determined in this case; the observed improvements occurred in the context of multimodal therapy. Fifth, despite genetic confirmation, the absence of cerebrospinal fluid (CSF) neurotransmitter metabolite assays precludes comprehensive characterization of the patient’s monoaminergic profile. Given that TH deficiency affects the biosynthetic pathways of catecholamines (dopamine and norepinephrine) while serotonin synthesis remains relatively preserved, measurement of homovanillic acid (HVA), 3-methoxy-4-hydroxyphenylglycol (MHPG), and 5-hydroxyindoleacetic acid (5-HIAA) in CSF would facilitate comprehensive assessment of dopaminergic, noradrenergic, and serotonergic functional status. The resulting HVA/5-HIAA ratio may provide insight into the relative imbalance between catecholaminergic and serotonergic systems, potentially elucidating the neurochemical basis of psychotic symptoms in THD.

## Conclusion

This case highlights the diagnostic and therapeutic challenges in managing psychotic symptoms in patients with THD. To the best of our knowledge, this represents the first reported case of genetically confirmed THD-associated dopa-responsive dystonia (DRD) with schizophrenia-like features, and is among the few documented instances of rTMS use in a patient with THD. The combination regimen of aripiprazole, quetiapine, and low-frequency rTMS was associated with clinical improvement in auditory hallucinations and delusions (based on clinical observation in the absence of standardized quantitative assessments), without exacerbation of dystonia (based on clinical observation), offering a potential therapeutic strategy for this vulnerable population. Clinicians should maintain a high index of suspicion for psychotic disorders in THD patients and exercise caution in balancing dopaminergic and antidopaminergic therapies. Further studies are warranted to establish optimal treatment protocols for this rare condition.

## Data Availability

The original contributions presented in the study are included in the article/supplementary material. Further inquiries can be directed to the corresponding author.
